# Antifungal Policy and Practice Across Five Countries: A Qualitative Review

**DOI:** 10.3390/jof11020162

**Published:** 2025-02-19

**Authors:** David W. Denning, John R. Perfect, Neda Milevska-Kostova, Artes Haderi, Hannah Armstrong, Maarten C. Hardenberg, Emily Chavez, Bruce Altevogt, Patrick Holmes, Jalal A. Aram

**Affiliations:** 1 Manchester Fungal Infection Group, The University of Manchester, Manchester Academic Health Science Centre, Manchester M13 9PL, UK; 2 Division of Infectious Diseases, Department of Medicine, Duke University School of Medicine, Durham, NC 27710, USA; perfe001@mc.duke.edu; 3 Department of Medical Microbiology, Radboud University Medical Center, 6525 GA Nijmegen, The Netherlands; neda.kostova@radboudumc.nl; 4 Patients for Patient Safety Observatory, Rue de Chantepoulet 10, 1201 Geneva, Switzerland; 5 Charles River Associates, London EC2M 7EA, UK; ahaderi@crai.com (A.H.); harmstrong@crai.com (H.A.); mhardenberg@crai.com (M.C.H.); 6 Pfizer Inc., New York, NY 10001, USA; emily.chavez@pfizer.com (E.C.); wayne.patrick.holmes@gmail.com (P.H.); 7 Biomerieux, 100 Rue Louis Pasteur, 69280 Marcy-l’Étoile, France; bruce.altevogt@biomerieux.com; 8 Melinta Therapeutics, Parsippany, NJ 07054, USA; jalalaram333@yahoo.com

**Keywords:** invasive fungal infections, surveillance, policy, stewardship, education

## Abstract

The burden of invasive fungal infections (IFIs) is increasing worldwide. National, regional, and local policies on IFI management should respond to the changing landscape. We assessed antifungal policies from five countries of varying size, IFI burden, and geography: the Netherlands, Italy, South Korea, China, and India. These countries were selected as a representative sample reflecting different types of economic and health systems that patients and providers access worldwide. This assessment focused on a comprehensive range of antifungal policy elements, including recognition and prioritization, awareness and education, prevention and monitoring, diagnosis and coordinated care, access to appropriate treatment, and diagnostic and treatment innovation. Although countries in this analysis all have some form of policy for IFI management, we have identified substantial gaps, including low prioritization of IFI diagnostics, omission of fungal pathogens from antimicrobial resistance policies, and a general lack of awareness and healthcare professional (HCP) training on IFI management. The gaps identified are intended to inform HCPs and policy- and decision-makers about aspects to consider in reducing the IFI burden for patients and health systems while demonstrating responsible antifungal stewardship.

## 1. Introduction

Each year, an estimated 150 million people experience serious fungal infections, with prevalence increasing worldwide [1]. A serious fungal infection is one that is life-threatening or associated with significant morbidity. Invasive fungal infections (IFIs) are systemic infections that are a major cause of morbidity and mortality usually in immunocompromised patients [2]. Globally, more than 2.5 million people die each year as a result of invasive fungal infections. The risk of IFIs is highest in critically ill or immunocompromised patients, including those with HIV/AIDS, poorly controlled diabetes, significant lung disease, transplant recipients, and people with cancer [3,4,5,6]. Chronic fungal infections cause considerable morbidity and can be fatal [7]. Despite this alarming public health problem, there remains a lack of clarity and recognition about the global burden of IFIs [1,8].

The main fungal pathogens responsible for the majority of cases of severe fungal disease are *Aspergillus*, *Candida*, and *Cryptococcus* species and *Pneumocystis jirovecii* and endemic dimorphic fungi such as *Histoplasma capsulatum*, as well as the emerging pathogenic molds, Mucormycetes [1,9]. The epidemiology of fungal diseases is dynamic—unexpected outbreaks of rare fungal diseases, epidemics involving established fungal pathogens, and climate change adaptation mechanisms in zoonotic fungi are difficult to predict [10].

Multiple clinical guidelines for different fungal diseases have been published, primarily for the management of affected patients but also for diagnosis. Some guidelines are for specific entities, while some have been developed at a national, regional, and, more recently, global level, especially for rarer IFIs [11]. In addition, there are many infection control guidelines published, some of which specifically include guidance on the prevention of IFIs (notably invasive candidiasis and aspergillosis) [12,13]. Few countries have health policies covering aspects of antifungal prescribing, diagnosis, monitoring, prevention, treatment, education, and stewardship of IFIs. We set out to investigate the current state of antifungal policies in a small sample of countries that represent a cross-section of global economic and health systems.

IFIs are associated with substantial costs to the healthcare system. In 2017, IFIs were associated with an estimated USD 7.2 billion in direct costs to the United States healthcare system, with *Candida* and *Aspergillus* infections accounting for the most hospitalizations and the highest total costs of any IFI [14]. In Europe, IFIs in cancer patients have been associated with additional costs ranging from approximately EUR 11,000–26,000 per patient [15]. Hospitalized patients with IFIs incur significantly higher costs compared to those unaffected [15].

Recent developments in molecular diagnostic assays have transformed the landscape of diagnosis, allowing for rapid identification. However, the use of molecular diagnostics is not yet widespread, leading to missed diagnoses, delays in treatment, and sometimes fatal outcomes [16]. Policy decisions with regard to diagnostic services tend to be primarily driven by economic factors, with less regard for local, hospital-based decision-making.

To ensure the appropriate diagnosis and treatment of IFIs, it is essential that healthcare professionals (HCPs) are knowledgeable about fungal diseases and their ongoing significant impact on human health [10]. Amid a lack of research on familiarity with IFIs, there have been recent calls to increase awareness among the public and physicians, with the goal of preventing delays in diagnosis and developing better treatments [10,17]. Some efforts in the field are contributing to improved IFI awareness, such as the inclusion of antifungals in the World Health Organization (WHO) Model List of Essential Medicines as well as the addition of several diagnostics (e.g., *Aspergillus* antigen and *Pneumocystis polymerase* chain reaction testing) in the WHO Essential Diagnostics List [18,19]. This list is updated and published every two years based on disease prevalence and public health relevance. In addition to previously published frameworks—including the Global Action Plan on Antimicrobial Resistance (GAP AMP), the Bacterial Priority Pathogens List, and the WHO Access, Watch, Reserve (AWaRe) Classification Database—the WHO recently issued a Fungal Pathogen Priority List to help draw attention to the need to improve the availability of treatments and diagnostics for fungal infections [20]. Despite efforts to improve awareness, there remains a lack of effective antifungal policies [21]. Thus, there is a need to map current guidelines and policies to understand gaps in the national policy landscape for IFIs to ensure patients have the best available care.

The objective of this review is to identify and communicate gaps in national and global antifungal policies by assessing the strengths and weaknesses of IFI-related policy landscapes from five example countries to raise awareness of key needs and issues in IFI prevention, diagnosis, and management, including antifungal access and development for the benefit of patients.

## 2. Materials and Methods

### 2.1. Framework Development Methodology

**Figure 1:**
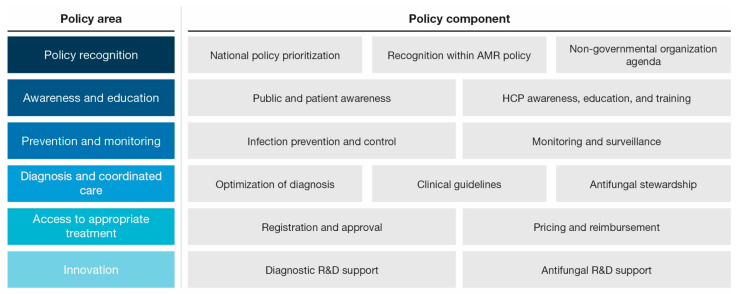
Framework of policy analysis (AMR—antimicrobial resistance; HCP—healthcare provider; R&D—research and development).

To analyze antifungal policy across selected countries, we developed a framework of six key areas of policy that are important to assess throughout the clinical journey for patients with IFIs. The framework aimed to support determination and articulation of the current landscape, policies, and other provisions in place to reduce the burden of IFIs and support patient care. To cover the policy environment top-down and move chronologically through the patient care journey, the following policy areas were selected: policy recognition, awareness and education, prevention and monitoring, diagnosis and coordinated care, access to appropriate treatment, and innovation. Each policy area was subdivided into two or three predefined policy components. The framework policy areas and the associated policy components are shown in Figure 1. The framework development process is described in the supplementary methods (Supplementary Materials).

The framework was applied to five selected case countries: The Netherlands, Italy, South Korea, China, and India. These countries were selected to provide a representative sample of different types of health systems worldwide and, thus, represent different geographical regions, levels of economic development, and population/size. All of the countries assessed have a high or growing burden of IFIs (either prevalence or rate of resistance) [22,23,24,25,26]. Additionally, these countries have geopolitical differences that may provide variety in political responses to managing IFIs but will also capture consistent end principles that identify local burdens and needs.

### 2.2. Literature Review Methodology

For each country, research was drawn from two main sources: (1) desk-based research focused on policies at both regional and national levels across all areas of the framework; (2) learnings from previous desk-based research focused on AMR policy and data on file. The research was conducted from January to May 2021.

A systematic approach was used to ensure a comprehensive review of both the published and grey literature. Searches were performed in databases including PubMed, Scopus, and Google Scholar. Keywords were derived from the framework policy areas and components (e.g., recognition, awareness, prevention, diagnosis, treatment, and innovation) and combined with terms such as ‘invasive fungal infections’, ‘antimicrobial resistance’, and ‘policy’. The grey literature sources were identified through government websites, think tank reports, conference proceedings, and media publications.

Inclusion criteria focused on studies and reports published between January 2016 and May 2021 that addressed antifungal policy, stewardship, surveillance, diagnosis, or treatment. Non-English sources and documents were translated using DeepL translation software (DeepL SE, Cologne, Germany). Data were extracted using a structured template aligned with the predefined framework categories, enabling systematic organisation and analysis. Screening and data extraction were performed independently by two reviewers, and discrepancies were resolved through consensus.

Data analyses drew from regional and national policies from the countries’ governments, as well as initiatives and proposals from patient organizations, think tanks, and specialists such as HCPs and academics. Information was gleaned from original documents published by the aforementioned stakeholders and organizations, as well as from the grey literature (including, but not limited to, websites, press releases, media and news articles, newsletters, and conference presentations).

### 2.3. Scoring and Validation Framework

Findings for each country across relevant framework policy areas in the form of a matrix table were then critically assessed to determine their level of development. Policy assessments were scored using a qualitative traffic light system (green, yellow, red) based on predefined criteria within each framework area. The scoring criteria were based on indicators assessing the presence, comprehensiveness, and degree of implementation of policies:

- **Green** indicates well-established policies with comprehensive implementation and monitoring.
- **Yellow** reflects either partial implementation or gaps in policy design.
- **Red** denotes either no formal policies or severe limitations in implementation.

Initial scores were drafted by the authors based on the literature and grey data reviews and then refined through focus group input (see below). Final scores were determined by majority consensus among authors, ensuring alignment with focus group insights and the literature findings.

A detailed description of the indicators and thresholds for each category is provided in Supplementary Figures S1 and S2 which also outline traffic light assessments for each policy component, including access to treatment and innovation, and what a well-developed policy framework should probably contain.

Findings were also reviewed with local Pfizer subject matter experts from each country; all experts were Pfizer employees and represented different business functions (public affairs, medical, market access, and commercial) in each country. Five semi-structured focus group discussions were held in July 2021 (one per country, 60 min duration), with two to five representatives per country. During these, representatives reviewed the compiled research by framework category and had an open discussion in which they provided their opinion about the comprehensiveness of existing policies and what governmental policy intervention would make the most impact on improving patient care in mycology. All findings were then reviewed and sense-checked by the authors for accuracy.

The same approach was used for all five countries: findings for each country were mapped to the relevant framework policy areas in the form of a matrix table and then critically assessed to determine their level of development.

## 3. Results

### 3.1. Policy Recognition

**Figure 2:**
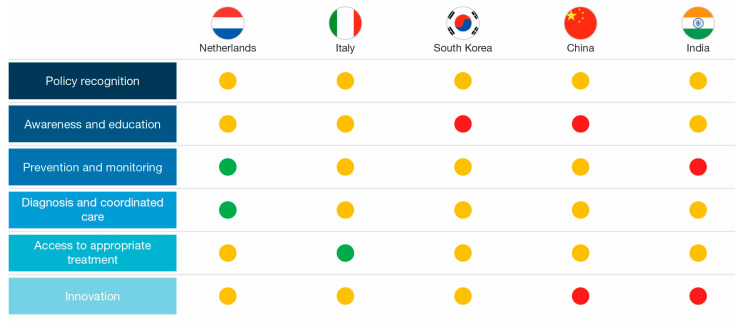
Summary of research findings. Green indicates established policies/initiatives AND/OR evidence of policy/initiative implementation. Yellow indicates that policies/initiatives exist but are limited in scope or timeframe, OR there is a lack of policies/programs currently but evidence of future developments. Red indicates a scarcity of current and future policies/initiatives.

We assessed the degree to which each of the five countries recognizes IFIs through the development of either government or non-government policies. We found that the level of recognition and prioritization placed on IFI policies by the different countries varies (Figure 2; Table 1). However, none of the countries demonstrated a robust policy (defined as formal governmental and non-governmental recognition of IFI burden and evidence of national policy recognition, including recognition within AMR policy) for the recognition and prevention of IFIs, and none prioritized IFIs or antifungal use in their national health policies.

In The Netherlands, the burden of IFIs is generally well recognized by the Dutch National Institute for Public Health and the Environment (RIVM) and academic hospitals [27,28,29], and a summary estimate was published in 2020 [24]. Annual AMR reports, which include IFIs, are produced jointly by the RIVM and the Dutch Working Party on Antibiotic Policy (SWAB) [30]. However, the prioritization of IFIs or antifungal use in national policies is limited.

In Italy, despite official policy recognition by the Italian Ministry of Health and the inclusion of antifungal drugs in the national AMR policy, there is limited academic work and data collection with regard to the national burden of IFIs [31,32], but again, a summary estimate was published in 2018 [23]. Prioritization of IFIs and antifungal use in national policies remains limited.

In South Korea, the current burden of IFIs is unclear, with only one identified study providing an estimate of the number of people affected every year, and there are no dedicated national policies on fungal infections [22]. AMR is generally well recognized, studied, and supported by government and government-affiliated research and policy initiatives (Table 1).

**Table 1:** Summary of policy recognition.

	The Netherlands	Italy	South Korea	China	India
National IFI policy	Y	Y	N	N	N
Government department responsible for AF policy	RIVM	Italian Ministry of Health	KCDC		Ministry of Health and Family Welfare/Ministry of Health/state governments
Nature of government policy	Advised centralized surveillance of *Aspergillus* resistance and infections	Aspergillosis and candidiasis on Infectious and Parasitic Disease list [31] IFIs recognized as serious complication of HIV/AIDS (2016) [25] IFIs recognized as serious risk for patients receiving chemo/radiotherapy (2018) [33]			Plans since 2016 to include resources for common fungal diseases at all levels of healthcare, including free AF medication for patients below the poverty line [34]
Omissions from government policy	No national policy action around at-risk patient groups	IFIs not part of 2020–2025 National Disease Prevention Plan [35]	No dedicated division for IFI, unlike viral and bacterial diseases (IFIs included in broader division of AMR)		2017 National Health Policy does not refer to IFI [36]
AMR policy	Y (RIVM, ARSWG)	Y (PCNAR)	Y	Y (NAP-AMR)	Y (NAP–AMR)
IFI/AF included in AMR policy	Y	Y	N	N	N
Nature of AMR policy	Yearly report led by RIVM and SWAB on AMR and AM use [25]		AMR management plan 2016–2020 focus on reduced usage		
IFI Surveillance/database	Y	N	N	Y	N
Nature of surveillance	Aspergillus Resistance Surveillance Working Group (non-government) supports science and research [37]		KCDC study into AF resistance in *Candida* as part of wider initiative on AF resistance research [38]	Chinese National Fungal Diseases Surveillance System, government-based, 2019 records resistance	ICMR launched the AMRSN for six bacterial and fungal species in 2013, now 30 labs across India Collaboration between National Mycology Reference Laboratory in Chandigarh and WHO is now nodal center for AF resistance surveillance [39]
NGO activity	Y	Y	N	N	Y
NGOs	Mycology Study Group, Radboudumc, Patient advocacy organizations	Italian Society for Anti-Infective Therapy (SITA)			Indian Association of Medical Microbiologists, Indian Council of Medical Research, GAFFI
Nature of NGO activity	Advocate awareness of influenza-related IA [6]	Raised awareness of influenza-related IA; encouraged national registries			Recommend creation of AF policy

AF = antifungal. AM = antimicrobial. AMRSN = Antimicrobial Resistance Surveillance and Research Network. ARSWG = Aspergillus Resistance Surveillance Working Group. GAFFI = Global Action Fund for Fungal Infections. IA = invasive aspergillosis. ICMR = Indian Council of Medical Research. IFI = invasive fungal infection. KCDC = Korean Centre for Disease Control. NAP-AMR = National Action Plan on AMR. NGO = non-governmental organization. PCNAR = National Contrast Plan of Antimicrobial Resistance. RIVM = Dutch National Institute for Public Health and the Environment. SWAB = Dutch Working Party on Antibiotic Policy. WHO = World Health Organization.

The Chinese government does not currently recognize fungal infections within national health policies or strategies, including the National Plan to Contain Antimicrobial Resistance. The Chinese National Fungal Diseases Surveillance System was established in 2019 and will take time to develop [25,40], with one comprehensive estimate published in 2020 [25].

In India, the burden of IFI is recognized by the Indian Ministry of Health and state governments. A surveillance program was launched in 2013, which tracks six pathogenic bacterial and fungal species and involves over 30 laboratories nationwide [41]. Despite this awareness, there is no national policy on IFIs across the country and India’s 2017 National Action Plan on AMR lacks mention of antifungal resistance [42]. A comprehensive burden estimate was recently published [26].

### 3.2. Awareness and Education

We assessed country-wide efforts to educate the general public/patients and HCPs on IFIs, as well as levels of awareness among each group. It was important to evaluate the availability of public information, as well as identify what type of formal training, if any, HCPs in each country receive on IFIs. A summary of information resources and education available to the public/patients and HCPs on IFIs is shown in Table 2.

Regarding HCP education and awareness overall, mycology is not focused on as part of most medical curricula or residencies/clinical rotations in any of the countries we assessed (Table 2). HCPs well versed in IFIs have generally “learned on the job,” utilized small-scale training programs, or specialized in certain areas, such as intensive care, infectious disease, or oncology. National professional societies, hospitals, and international scientific societies offer educational online and onsite courses and workshops with the aim of enhancing the knowledge and skills of individuals working in the field of mycology; groups such as the European Society of Clinical Microbiology and Infectious Diseases, the British Society for Antimicrobial Chemotherapy, and the International Society for Human and Animal Mycology [49].

In the Netherlands, there is minimal formal training on IFIs in the Dutch HCP general medical curriculum guidelines, and post-education resources such as a course on *Aspergillus* and azole resistance and Radboudumc-sponsored SWAB guideline seminars are utilized [50].

Of the countries analyzed, Italy appears to have the most training resources for HCPs on IFIs. Specialist centers and non-government training initiatives, such as those from the Italian Society for Microbiology, organize HCP training on new antifungals and ways to optimize therapy [51,52]. Additionally, the hematology community is developing specific treatment guidelines for IFIs, but these are not formally published as yet.

In South Korea, the academic literature on management and prevention options is available, yet HCPs generally rely on US-based guidelines and reports from the Infectious Diseases Society of America (IDSA) [47,48,53,54].

In China, there is a current lack of widely available IFI education programs for HCPs led by government or non-government organizations; however, the Global Action for Fungal Infections has been working to organize training workshops and meetings on medical mycology.

Similarly, in India, most HCPs do not receive formal training on recognizing and managing fungal infections, though ad hoc education does occur. For example, in May 2021, the Press Information Bureau of the Indian Government and the Ministry of Health and Family Welfare produced an information package to the general public on the severe, fatal outbreak of mucormycosis in patients with COVID-19 within two days of announcing the outbreak. This represents a rapid, reactive response during a severe surge in this invasive mycosis [55], which, whilst positive, also highlights the lack of proactive educational efforts ahead of such a severe outbreak.

### 3.3. Prevention and Monitoring

We assessed the existence of policies to support the prevention and monitoring of IFIs. We found that the level of prevention and surveillance strategies varied across nations, from a generally decentralized approach (the Netherlands and India) to more limited centralized efforts (Italy and South Korea; Table 3).

Monitoring and surveillance are decentralized in the Netherlands, and although plans exist for a centralized approach, there is no evidence that this has been implemented [27]. Nevertheless, SWAB guidelines recommend robust prevention-driven approaches for at-risk populations, and Dutch hospitals apply advanced hygiene measures driven by outbreaks or policy changes [56,57].

In Italy, there is evidence of an organized *Aspergillus* Intensive Care Unit sample collection and tracing effort, as well as prevention guidelines for aspergillosis set up by the National Institute of Infectious Diseases Lazzaro Spallanzani (NIIDSS) [67]. Hospital hygiene and infection prevention are suboptimal; however, the government has recently taken action to improve hygiene standards.

In South Korea, monitoring and surveillance efforts to understand the burden of IFIs are limited to the use of claims data from the National Health Insurance Service (NHIS) [60]. A national policy exists on infection and prevention in hospitals, although it is not specific to IFIs [68]. Prevention-driven policies are absent, but an increase in the use of antifungal prophylaxis has been observed in recent years [69].

In China, the Chinese National Fungal Diseases Surveillance System was established in 2019 [25,40]. Other sources of IFI monitoring data are also available; however, infection prevention and control practices for IFIs are variable across regions and hospitals, with no clear national policy.

The Indian healthcare system does not have a centralized surveillance and monitoring program for IFIs. In 2015, multiple governmental and private authorities expressed concern that a nationwide IFI surveillance system was needed, but there is as yet no outcome from those statements [65,70]. Smaller-scale projects have been undertaken; for example, in response to a surge in cases of coronavirus disease-associated mucormycosis in June 2021, an online collaborative registry was developed [71].

### 3.4. Diagnosis and Continued Care

Timely diagnosis of IFIs is critical for effective treatment, and appropriate stewardship efforts may help improve treatment efficacy. Properly diagnosing IFIs is dependent on access to diagnostic laboratories, laboratory capacity, local HCP expertise in IFIs, and availability of rapid diagnostic tools, among other factors. We sought to evaluate some of those factors (Table 4).

Diagnostic capabilities in the Netherlands vary between treatment centers; however, expert support for IFI diagnostics and testing is available through The Center of Excellence on Fungal Infections at the Radboud University Medical Center [43]. Comprehensive clinical guidelines (SWAB Management of Invasive Fungal Infections guidelines) have been established to cover multiple IFIs and are updated every five years or when significant scientific advances around IFIs have been made [58]. SWAB Antimicrobial Stewardship guidelines also cover fungal pathogens driven by high azole resistance in the Netherlands, and each hospital has an Antimicrobial Stewardship Team (A-team) to implement and monitor adherence to the SWAB guidelines [58,80].

In Italy, evidence suggests that there is a centralized approach to diagnosis, and Italian HCPs generally have access to advanced diagnostic techniques [83,84]. However, it is unclear whether there is a centralized policy approach to IFI management and antifungal stewardship despite non-government consensus guidelines and small-scale stewardship initiatives. Although Italy does not have a centralized clinical guideline for IFI management, there are a host of resources available for Italian HCPs, and the Italian Antimicrobial Stewardship Group has been involved in specific stewardship guidelines for candidemia infections [61,62,72,74,81,85].

South Korea has centralized diagnostic capabilities for IFIs, and a high proportion of the hospitals have advanced diagnostic technologies [77,78,86,87]. However, comprehensive and centralized guidelines appear to be lacking, and there is no evidence of recent or ongoing antifungal stewardship programs.

In China, access to rapid, advanced diagnostic testing for IFIs is limited [76,79]. China has centralized clinical guidelines on IFIs; the Chinese Invasive Fungal Infection Working Group developed a consensus diagnosis and treatment guideline in 2005 [75,88]. Unfortunately, many HCPs in public hospitals cannot adhere to the guidelines due to reimbursement restrictions and will instead follow IDSA and local country guidelines [76]. There is no evidence of a centralized antifungal stewardship program in China. These gaps in care provision were recently acknowledged in a summary of a national plan to combat fungal diseases [89].

In India, lack of access to rapid diagnostics and subsequent poor IFI management is of concern, although there have been efforts to improve IFI diagnostics through the establishment of diagnostic mycology laboratories [39,76,90,91]. Centralized clinical guidelines on IFI are not available, and the majority of Indian HCPs use the IDSA guidelines to manage IFIs [78]. The Ministry of Health and Family Welfare of the Government of India, together with academia, has launched several antimicrobial stewardship programs since 2010. Calls for improved diagnosis (and clinical awareness) have been partially heeded [92], and certainly, the COVID-19 pandemic provided a wake-up call with respect to mucormycosis [93]. However, the Indian Association of Medical Microbiologists believes that antifungal stewardship should be separated, as antifungal resistance is increasing in India [70].

Although some countries do not have clinical guidelines for the optimal diagnosis of IFIs, global guidelines do exist for some IFIs as part of the “One World One Guideline” initiative. This initiative, set out by the European Confederation of Medical Mycology, aims to diagnose IFIs earlier and facilitate a more targeted treatment strategy. Guidelines for mucormycosis, rare molds, endemic mycoses, rare yeasts, and COVID-19-associated pulmonary aspergillosis are already available and are applicable worldwide [11,94,95,96,97]. We were not able to track whether or how these guidelines were or were not implemented, although, for rarer infections, it is likely to be on a case-by-case basis and led by the local clinical team.

### 3.5. Access to Treatment

Medicine approval processes vary greatly among the countries we assessed. In countries that use health technology assessments in the decision-making process on reimbursement of a new drug, including the Netherlands, Italy, and South Korea, antifungal treatments are typically required to undergo clinical and economic assessment to gain reimbursement [98,99]. Similar to antibiotics, this is often difficult due to inappropriate comparators and data limitations and may limit access to novel antifungals [100].

Pricing and affordability represent one of the key barriers to IFI treatment for patients. In the Netherlands, prescription costs are reimbursed, but drugs may be substituted for others, and patients may have to pay the difference [101]. In Italy, the most recent antifungal drugs are reimbursed, indicating no specific barriers to treatment access at a national level, although regional delays to formulary inclusion have led to access delays in specific regions [102]. Similarly, in South Korea newer antifungals have been reimbursed since 2014 by the NHIS [103]. In China and India, the preferred choice of antifungal drug is not always the most affordable; thus, physicians are often prevented from prescribing their preferred choice [76]. In addition, some preferred antifungal drugs are not widely available in some Asian countries, which represents another barrier to improving the management of IFIs [76]. For example, anidulafungin is not available in China, and AmBisome™ has only recently been approved in China despite being a regularly used antifungal therapy option in Europe and the United States for decades.

### 3.6. Innovation

As of early 2023, only five major classes of antifungal agents (azoles, polyenes, flucytosine, echinocandins, and allylamines) have been used to treat IFIs, with three recently approved agents in the United States (otesaconazole, ibrexafungerp, and rezafungin) [104,105,106]. There are currently only two systemic antifungal agents in late-stage development for IFIs: olorofim and fosmanogepix [107]. Although the countries we assessed vary in terms of the amount of research and development ongoing in the areas of IFI diagnostics and treatment, there is overall a limited amount of support for the development of novel diagnostics and treatments within these countries. The Dutch government, by way of RIVM, has set up a system (Nationaal Diagnostisch Vademecum Infectieziekten) for HCPs and microbiologists to identify diagnostic laboratories with the required expertise for infectious diseases, including IFIs [108]. The RIVM and Radboudumc have also initiated efforts to study resistant *Aspergillus* [109,110]. However, there is no evidence to show that the development of novel diagnostics is supported nationally. Despite limited government funding, recent publications by Italian universities suggest increased research activity in the IFI field [111,112,113]. IFI diagnostic innovation in South Korea is also largely driven by academia, but there is some evidence that the government (Center for Infectious Disease Research) is interested in supporting innovative efforts to understand antifungal resistance and develop new medicines [114]. There is no evidence that the Chinese government supports IFI diagnosis innovation. Government support for treatment innovation is also limited, but external sources, such as the United Kingdom Department of Health, have provided some funding to improve antifungal innovation [115]. Although India has made an effort to improve its infection disease diagnostics, there is no evidence of diagnostic innovation in India, and IFIs were largely not included in the 2015–2020 or 2021–2025 strategies [39,116,117]. Similarly, there is no evidence that India is currently supporting novel antifungal treatment development domestically.

## 4. Discussion

The results of this review highlight best practices and areas for improvement in global, regional, and local antifungal policy (Figure 2). Given the increasing global burden of IFIs, alongside the notable gaps in policy aspects across a sample of countries, there is an urgent need to update and revise, or create, fungal infection diagnosis and treatment guidelines, prioritize antifungal stewardship, and dedicate resources to improve access to rapid diagnostic and treatment options, particularly in high-risk populations and high-burden areas.

The comprehensive framework used in this research was developed with the patient care journey in mind. Similar frameworks have been used to analyze policies in other diseases, including breast cancer and rare diseases [118,119]; however, such a framework has not yet been utilized for infectious diseases. Thus, the framework developed in this study represents a novel approach to assessing the current policy environment for patients with IFIs.

To improve antifungal policy in the countries assessed and others across the world, it is important to understand what an ideal policy on fungal diseases would include. For example, national, regional, and hospital policies for fungal diseases should all be considered, and each of these policies should include diagnostic, surveillance, antimicrobial therapy, stewardship, and educational elements.

### 4.1. An Ideal IFI Policy: Diagnostics

With respect to diagnostics, the provision of rapid testing for fungal infection in immunocompromised and critically ill patients is fundamental, and healthcare systems should enable result turnaround times earlier than currently [120]. Routine screening for IFIs in immunocompromised and critically ill patients should also be considered. Of the countries assessed, HCPs in the Netherlands, Italy, and South Korea appear to have the highest access to rapid, state-of-the-art diagnostics, although some diagnostic facilities are centralized, thereby increasing the time to diagnosis. A survey to assess the competencies of mycology laboratories in Asian countries, including China and India, was conducted by the Asia Fungal Working Group. Findings from this survey indicate that mycology laboratories across Asia are often not accredited, and many lack access to advanced non-culture-based diagnostic tests [121]. Further, in China, the lack of rapid testing facilities is cited as a key reason for suboptimal IFI management [79]. The diagnostic capacity of IFIs across Europe is more developed, as most institutions are capable of processing isolates and have access to susceptibility testing; however, resources for the diagnosis and management of IFIs vary depending on the gross domestic product of the country [122]. For example, a successful diagnostic facility is in Guatemala, where a program was set up to improve diagnosis and care of IFIs in patients with HIV through coordinated actions that comprised enhanced ‘free’ diagnostics in HIV units, a rapid sample transport system, an online ordering and results system, as well as extensive staff training; the program has since expanded and Guatemala now has the first Diagnostic Laboratory Hub that specializes in mycology and provides diagnostic services to patients with HIV across Central America, with evidence generated intended to justify fungal disease strategies in the national health program [123]. Unfortunately, recognition of at-risk patient populations is low among all the countries we assessed, as those populations are often not included in national or regional policies. High-quality, rapid, and extensive diagnostic services contribute to the global control of AMR and will continue to do so, as ruling in or out fungal diseases allows for the discontinuation of unnecessary antibacterial or antifungal therapy and the appropriate direct therapy to be administered [124].

COVID-19-associated fungal infections have highlighted the need for increased awareness and improved diagnostic strategies for IFIs. In India, a surge of mucormycosis cases recently occurred in patients with COVID-19, uncontrolled diabetes, and systemic corticosteroid treatment identified as risk factors [125,126]. For example, in India, several guidance documents are now available regarding COVID-19-associated mucormycosis, with glycemic control and corticosteroid management included in most guidelines [127,128,129].

### 4.2. An Ideal IFI Policy: Prevention and Monitoring

Surveillance efforts should include monitoring of fungal burden, resistance, and efficacy of prophylaxis and early empiric therapy protocols. This would enable early detection of potential outbreaks linked to lapses in infection control and antifungal resistance and inform future protocols. Our research identified surveillance systems in each country, with China and Italy having fungal surveillance efforts in place and the Netherlands, Italy, and India monitoring resistance for selected fungal pathogens. Large-scale surveillance studies have been published since the establishment of the Chinese National Fungal Disease Surveillance System, and a published independent study has shown the benefit of prophylaxis, highlighting the importance of centralized monitoring through fungal surveillance systems [63,66]. AMR policies in South Korea, China, and India focus primarily on bacterial resistance. The Netherlands and South Korea have an increased focus on the use of antifungals prophylactically, but it is not clear how effective these efforts have been at reducing the IFI burden.

### 4.3. An Ideal IFI Policy: Coordinated Care

In an ideal clinical antifungal policy, specific guidance on initial and second-line antifungal treatment options should follow the latest national and international evidence base. Infectious disease consultation for both complex and life-threatening fungal infections should also play a role in the development of this guidance. Systems should be established to also ensure periodic review of guidelines to confirm that they reflect the current science. Our research showed that only the Netherlands has centralized guidance on the management of IFIs. Italy has a number of independent resources available for fungal disease management, but HCPs in South Korea, China, and India rely on US-based guidance despite barriers to accessing the medication and diagnostics recommended in that guidance.

### 4.4. An Ideal IFI Policy: Stewardship

Antimicrobial stewardship should link negative diagnostic test results with the discontinuation of unnecessary antibacterial, antiviral, and antifungal therapy and should provide expert guidance for problematic drug–drug interactions. Therapeutic drug monitoring approaches for antifungals with variable pharmacology should help inform stewardship efforts. Our research found that only the Netherlands has a centralized antifungal stewardship initiative, with a custom, localized version of the online national antimicrobial guide available to every hospital and an A-team in each hospital [58]. Italy has some decentralized initiatives, while South Korea, China, and India have limited or no antifungal stewardship despite increasing antifungal resistance and IFI burden.

### 4.5. An Ideal IFI Policy: Awareness and Education

Educational programs should be overarching and continuous to increase awareness of fungal disease among all healthcare workers, including early consideration of fungal disease in formulating differential diagnosis; key diagnostic data, resistance trends, and antifungal therapy performance should be incorporated. Our research indicates that public and HCP awareness of IFIs is very low across all the countries we assessed. Although efforts have been made to enhance the general awareness of IFIs, such as the recently issued WHO Fungal Priority Pathogens List [20], major improvements are still required. For example, the 2017 European Union’s One Health Action Plan against AMR and the 2014 Asia-Pacific Economic Cooperation (APEC) Guideline to Tackle AMR in the Asia-Pacific Region are mainly concerned with bacteria and antibacterial stewardship, with sparse mention of fungal pathogens and resulting infections [130,131]. While global awareness of IFIs may be increasing with the emergence of COVID-19, HCP training programs need to be expanded to include IFI management across more medical specialties.

The research carried out to complete this review was thorough and robust, covering multiple aspects of antifungal policy encompassing government initiatives, awareness, diagnostics, treatment, surveillance, and stewardship. We utilized desk research, the grey literature, and “on-the-ground” local expertise primarily due to the general lack of awareness and policy around IFIs preventing any meaningful systematic literature review. As such, we structured the research by framework and applied quasi-systematic research within each of the framework’s elements. We also note that the nature of our interviews with local experts was subjective, and interviewees may not have been completely forthcoming about the state of policies around IFI management in their home countries. More information could have been gathered from additional interviews with patients, local HCPs, representatives from non-governmental organizations, or local policy-makers. We selected countries with the aim of covering a broad range of socio-political and socio-economic populations, but we did not consider African, North American, Oceanian, and South American populations in this analysis. As countries have varying needs, policies will need to be localized appropriately.

### 4.6. Limitations and Reproducibility

The methods described were designed to ensure transparency and reproducibility. However, generalisability of focus group findings may be limited due to the pharmaceutical industry-focused perspectives of participants. Future studies may consider incorporating broader stakeholder groups, including healthcare professionals and policymakers. While the Delphi methodology offers advantages for building consensus, focus groups were chosen to provide real-time qualitative insights and contextual validation of preliminary findings.

## 5. Conclusions

IFIs are a substantial and growing global health problem, and effective treatment options are limited and waning, even in regions of the world where they are readily available due to antifungal resistance. Notable gaps across global, regional, and national policies contribute to this worsening burden of IFIs. Current gaps include omission of fungal pathogens from most AMR policies, poor prioritization of IFI diagnostics and antifungal research and development, low IFI awareness among HCPs and the public, a lack of formal HCP training programs on IFIs, and poor access to affordable treatment. The Netherlands, Italy, and South Korea have strong aspects to their IFI policies and infrastructure, such as robust management guidelines in the Netherlands, a dense population of IFI experts in Italy and stringent prevention protocols in South Korea. However, the burden of IFIs in these countries is also lesser known and public awareness of IFIs is low. In contrast, the burden of IFIs in China and India is higher, but policies and guidelines on IFI management and resources to diagnose and effectively treat IFIs in those countries are limited.

An ideal policy should include diagnostic, surveillance, antimicrobial therapy, stewardship, and educational elements. Developing effective antifungal policies at local, regional, and global levels and expanding upon existing policies remain critical to improving the management of IFIs and alleviating the burden for the estimated 150 million people who experience severe fungal infections every year.

## Figures and Tables

**Table 2:** Summary of awareness and education.

	The Netherlands	Italy	South Korea	China	India
Nature of public awareness efforts	National expert center website through Radboudumc [43] Some national TV/media channels	Some medical websites only ECDC visit to increase public awareness of AMR (2017) [44]	Hospital and public health websites Awareness campaign “Fungal Infection Awareness Week” * [45] Publicly available manual on aspergillosis	No evidence of awareness initiatives	Fungal disease awareness week, organized by US CDC [46]
IFI in HCP training curriculum	Y (primarily specialists)	Y (specialist only)	N	N	N
Educational tools for HCPs	HCP curriculum guidelines only Resources for post-education professionals (e.g., MSD course on *Aspergillus* and azole resistance and Radboudumc-sponsored SWAB guideline seminars)	History of industry-sponsored training Italian Society for Microbiology organizes training on new therapies Development of specific IFI treatment guidelines by Italian hematologists	Academic literature through Synapse database [47] US-based resources (e.g., IDSA guidelines) [48]	GAFFI-organized training efforts (medical mycology workshops and scientific meetings)	Fungal Infectious Study Forum training courses WHO SEARO training program Small, private initiatives (100 participants) National Mycology Reference Centre laboratory training

* World Fungal Infection Awareness Week is a global initiative; however, evidence of country-level activity was only found for South Korea. AMR = antimicrobial resistance; CDC = Center for Disease Control; ECDC = European Centre for Disease Prevention and Control; GAFFI = Global Action Fund for Fungal Infections; HCP = healthcare professional; IDSA = Infectious Diseases Society of America; IFI = invasive fungal infection; MSD = Merck Sharp & Dohme Corp; WHO SEARO = World Health Organization South East Asia Regional Office.

**Table 3:** Summary of prevention and monitoring.

	The Netherlands	Italy	South Korea	China	India
Specific IFI prevention strategy	N	N	N	N	N
Nature of infection prevention strategies	Advanced hygiene measures in hospitals, driven by outbreaks or policy updates [56,57] Advice from independent NGOs Internal hospital protocols [57] SWAB guidelines for at-risk populations [58]	NIIDSS Aspergillosis prevention guidelines Infection prevention protocols are lacking, generally Government action was taken in 2017, but unclear on the goals and outcomes	Ministry for Health and Welfare provided a comprehensive list of prevention measures in 2015, including outbreak response protocols and negative-pressure isolating rooms [59]	There is no evidence of organized prevention strategy	There is no evidence of organized prevention strategy
Centralized monitoring	N	Y (IA only)	N	Y	N
Nature of monitoring and surveillance	Azole resistance testing Surveillance system proposed in 2017, no evidence of action [27]	Surveillance other than ICU aspergillosis is poor No centralized system exists to map the burden of IFIs	Burden of IFIs is not easily assessed; must use claims data [60] Specialized Pathogen Resource Banks focus on fungal infections Fungal AMR has been well-monitored by academic institutions since 2017 [61,62]	Chinese National Fungal Disease Surveillance System created in 2019; no results yet [25] Various large-scale surveillance studies [63,64]	Integrated Diseases Surveillance Programme does not include fungal pathogens Surveillance stated as priority in 2015, but no evidence of action [65]
Status of prophylaxis measures	Extensive testing performed for at-risk populations		Recently increased focus on prophylactic therapies	Independent studies have been published showing benefit of prophylaxis [66]	

AMR = antimicrobial resistance; IA = invasive aspergillosis; ICU = intensive care unit; IFI = invasive fungal infection; NGO = non-governmental organization; SWAB = Dutch Working Party on Antibiotic Policy; NIIDSS = National Institute of Infectious Diseases Lazzaro Spallanzani.

**Table 4:** Summary of diagnosis and coordinated care.

	The Netherlands	Italy	South Korea	China	India
Centralized clinical guidelines for IFIs	Y	Unclear	N	Y	N
Nature of clinical guidelines	SWAB Management of Invasive Fungal Infections, updated every 5 years, covers invasive *Candida*, *Aspergillus*, *Cryptococcus neoformans*, and *Mucorales* [58] Custom, localized SWAB-ID guide available to every hospital Additional guidance for IA and COVID-19 was developed by the Expertisecentrum Schimmelinfecties Radboudumc together with NVALT	Guidance exists for IFIs in solid organ transplant and IC management [72,73] ECIL-6 provides guidance on IC, IA, and mucormycosis in leukemia and stem cell transplant patients [74]	HCPs generally use American Society of Infectious Diseases (IDSA) guidance	Chinese Invasive Fungal Infection Working Group guidelines are updated approximately every 4 years [75] Guidelines are not easy to adhere to in practice due to reimbursement Most physicians use IDSA or local country guidance	HCPs generally use IDSA guidance [76]
Diagnostic and treatment resources	Expert multidisciplinary consultation team at the Expertisecentrum Schimmelinfecties Radboudumc can assist with diagnostics and treatment planning	Dedicated diagnostic facilities at Italian National Institute of Infectious Diseases to identify fungi using PCR, fragment analysis, and gene sequencing HCPs have access to advanced diagnostic techniques (e.g., *Aspergillus* lateral flow tests, PCR testing kits) Dedicated clinic for patients with chronic pulmonary aspergillosis	Likely centralized diagnostic capabilities Some hospitals have advanced diagnostic techniques [77,78] Distribution of diagnostic technology limited	Access to rapid, advanced diagnostic testing for IFIs is scarce [79] Most hospitals have no facilities for mycology testing Hospitals tend to use culture-based diagnostics, although cryptococcal antigen, beta D glucan, and next-generation sequencing are readily available	Access to rapid, advanced diagnostic testing for IFIs is scarce [76] Onsite, hospital-based diagnostic facilities scarce National Mycology Reference Laboratory at PGIMER, Chandigarh ICMR supporting development of 40 nationwide mycology reference laboratories (2019–2025)
Nature of IFI stewardship	Azoles are included in the SWAB Antimicrobial Stewardship guidelines, driven by high azole resistance in The Netherlands [80] Each hospital has an AMS Team	PCNAR includes antifungal drugs and AMS [32] Italian AMS group has been involved in Candidemia stewardship guidelines [81]	No evidence of national AMS Country Cooperation Strategy 2019–2023 called for stewardship efforts [82]	No centralized stewardship Low prevalence of stewardship in individual hospitals [76]	Ministry of Health and Family Welfare of the Government of India has launched several stewardship programs since 2010 [70]

HCP = healthcare professional. IA = invasive aspergillosis. IC = invasive candidiasis. ICMR = Indian Council of Medical Research. IDSA = Infectious Diseases Society of America. IFI = invasive fungal infection. NVALT = Dutch Association of Chest Physicians. PCNAR = National Contrast Plan of Antimicrobial Resistance. PCR = polymerase chain reaction. PGIMER = Postgraduate Institute of Medical Education and Research. SWAB = Dutch Working Party on Antibiotic Policy. SWAB-ID = SWAB online national antimicrobial guide.

## Data Availability

No additional data is available—all sources are referenced.

## Supplementary Materials

The following supporting information can be downloaded at: https://www.mdpi.com/article/10.3390/jof11020162/s1, Figure S1, Scoring system used to evaluate different policies, projects, or initiatives; Figure S2, Indicators of a well-developed antifungal policy environment.
