# Supplementary material for: Antifungal Policy and Practice Across Five Countries: A Qualitative Review
Source: J Fungi (Basel). 2025 Feb 19;11(2):162. doi: 10.3390/jof11020162 (PMC11856614; doi:10.3390/jof11020162)
Supplement: Supplementary file 1 [file jof-11-00162-s001.zip › jof-3392706-supplementary.pdf]

Supplementary materials

1. Supplementary Methods

1.1 Framework development

The antifungal policy framework was designed to comprehensively address various aspects of antifungal therapies, ranging from awareness and recognition to patient access, treatments, and innovation. This multi-modal approach was chosen as it supported identifying strengths and weaknesses in the overall policy environment, which might be overlooked in a more quantitative framework [127].

At the same time, the individual pillars within the framework were chosen based on the significant importance of government recognition, awareness, and appropriate access to treatment when it comes to developing policy for infectious diseases. Within each pillar, we opted to include a wide range of policy components that would support broad investigation of the policy environment [128].

1.2 Scoring system

Our scoring system comprises a traffic-light system of appraisal (Figure S1), which was chosen as it provides a simple way to assess and communicate the progress, performance, or status of various policy initiatives or objectives. We sought to use a scoring system that provides a consistent approach to evaluating different policies, projects, or initiatives, to facilitate comparisons and benchmarking. This was particularly relevant given the wide variation in scope and depth of antifungal policies across countries. At the same time, highlighting areas of concern or underperformance helps decision makers focus their efforts and resources on the most pressing issues, leading to more efficient and targeted policy interventions.

Indicators of a well-developed antifungal policy environment are shown in supplementary figure (Figure S2).

Supplementary Figure S1. Scoring system used to evaluate different policies, projects, or initiatives.

	Level of antifungal policy development		
	High	Medium	Low
**Policy component**	• **Formal, established policy/initiative that is specific to antifungals**<br>• Scope of policies are comprehensive and well designed, with evidence of success<br>• Evidence of effective implementation of policies<br>• Uptake of policies are without large regional disparities	• Existence of policies/ initiative(s), but initiatives are limited in scope, timeframe and implementation.<br>• Lack of policies implemented currently, but clear evidence/ future (~next three years) initiatives to be implemented	• Lack of current and future (~next three years) initiatives/ efforts<br>• There is no debate to implement policies and programs in the future

Supplementary Figure S2. Indicators of a well-developed antifungal policy environment.

Level of policy development	Policy area					
	Policy recognition	Awareness and education	Prevention and monitoring	Diagnosis and coordinated care	Access to appropriate treatment	Innovation
	Formal governmental and non-governmental recognition of IFI burden: evidence of national policy action, including recognition within AMR policy	Evidence of public awareness of IFIs; access to formal HCP training/education with evidence of uptake; sufficient number of ID and/or IFI specialists	Establish national surveillance systems, with publicly accessible data which is used to inform policy; comprehensive hospital-level IPC guidelines with evidence of uptake	Timely access to diagnosis, with regularly updated diagnosis and treatment guidelines; high-quality integrated care between HCPs; growing uptake of antifungal stewardship	Timely registration/approval of antifungals; no major barriers to access to novel antifungals; valuation process for novel antifungals does not create major challenges	Government/non-governmental stakeholder support to develop new diagnostic tools and novel antifungals/IFI therapy (e.g. inclusion in AMR R&D incentives)
	Burden of IFI is informally recognized, and policy action is either minimal or lacking; recognition within AMR policy is minimal or lacking	Limited public awareness with no initiatives to improve this; limited HCP training/education programs, or evidence of limited uptake; insufficient number of ID specialists	Absent or limited national surveillance systems, with some ad hoc data reporting by academics (e.g. for key pathogens); limited or sporadic IPC measures at hospital level	Limited access to timely diagnosis (e.g. with delays); variable quality or infrequently updated diagnosis and treatment guidelines; sporadic implementation of antifungal stewardship	Delays in registration/approval of antifungals; some barriers to access of new antifungals (e.g. delays or restrictions in reimbursement); inappropriate valuation process for antifungals	Limited government/non-governmental stakeholder support to develop new diagnostic tools or novel antifungals/IFI therapy; missing link with AMR R&D support
	Burden of IFI is not recognized and national policy action does not exist; recognition within AMR policy is lacking	No evidence of public awareness; no formal awareness efforts or education initiatives in place, and insufficient HCP training/education including lack of ID and/or IFI specialists	No national surveillance systems and no academic data available for most pathogens; limited uptake of and accountability for IPC measures (e.g. in private hospitals only)	Severe challenges in obtaining timely diagnosis; no or low-quality diagnosis and treatment guidelines; evidence of poor antifungal prescribing practices	No timely registration/approvals of antifungals; reimbursement barriers or lack of broad access of new antifungals (e.g. significant affordability hurdles)	No government/non-governmental stakeholder support to develop new diagnostic tools or novel antifungals/IFI therapy; no link with AMR R&D support

AMR= antimicrobial resistance; HCP= healthcare professional; ID= infectious diseases; IFI= invasive fungal infection; IPC= infection prevention and control; R&D= research and development.
